# Microbial community shifts in deposits from the 2018 Palu–Donggala tsunami

**DOI:** 10.1093/ismeco/ycag114

**Published:** 2026-07-03

**Authors:** Wenshu Yap, Adam D Switzer, Jędrzej M Majewski, Winona Wijaya, Ezequiel Santillan, Rachel Y S Guan, Benjamin P Horton, Benazir Benazir, Ella Meilianda, Federico M Lauro

**Affiliations:** Earth Observatory of Singapore, Nanyang Technological University, 639798, Singapore; Earth Observatory of Singapore, Nanyang Technological University, 639798, Singapore; Asian School of the Environment, Nanyang Technological University, 639798, Singapore; Earth Observatory of Singapore, Nanyang Technological University, 639798, Singapore; Institute of Geology, Adam Mickiewicz University, 61606, Poznań, Poland; Asian School of the Environment, Nanyang Technological University, 639798, Singapore; Singapore Centre for Environmental Life Sciences Engineering, National University of Singapore, 117456, Singapore; Singapore Centre for Environmental Life Sciences Engineering, Nanyang Technological University, 637551, Singapore; Asian School of the Environment, Nanyang Technological University, 639798, Singapore; School of Energy and Environment, City University of Hong Kong, Hong Kong; Civil and Environmental Engineering Department, Faculty of Engineering, Universitas Gadjah Mada, Jl. Grafika, Kampus No.2, 55284, Yogyakarta, Indonesia; Tsunami and Disaster Mitigation Research Center, University Syiah Kuala, 23111, Banda Aceh, Indonesia; Asian School of the Environment, Nanyang Technological University, 639798, Singapore; Luminis Water Technologies, 619525, Singapore

**Keywords:** tsunami deposits, DNA, earthquake, coastal management

## Abstract

The 2018 Palu–Donggala earthquake in Indonesia generated a devastating tsunami, despite originating from a strike-slip fault, which typically does not produce significant tsunamis. This tsunami was triggered by a combination of subaerial landslides, liquefaction, and submarine landslides. Here, we present the first application of sedimentary environmental DNA to characterize microbial community changes in deposits from a landslide-triggered tsunami. Sediment samples collected 2 months after the event provide a rare snapshot of the near-immediate microbial response to tsunami disturbance. Microbial assemblages derived from 16S rRNA gene sequencing clearly distinguished tsunami deposits from pre-tsunami samples across three sites in Palu Bay, even where conventional sedimentary evidence was inconclusive. The community composition varied among sites, suggesting site-specific environmental filters, which explain the absence of a universal tsunami microbial signature. Tsunami deposits were associated with microbial communities shaped by strong environmental disturbance linked to saline inundation and geochemical change. Microbial communities in the Palu tsunami deposits provide new insight into early microbial responses to extreme coastal flooding events.

Tsunamis are among the most destructive natural hazards, and their generation mechanisms can be complex. The 2018 Palu–Donggala tsunami in Indonesia, generated by a strike-slip earthquake along the Palu-Koro fault and subsequent subaerial landslides [1], liquefaction [2, 3], and submarine landslides [4], represents a rare landslide-triggered tsunami event.

Tsunami deposits are commonly identified using sedimentological [5], geochemical [6], and/or microfossil indicators [7], but these methods can be ambiguous when preservation is poor or provenance is ambiguous [8]. Recent studies have shown that microbial community shifts preserved in sedimentary environmental DNA (sedDNA) can serve as sensitive indicators of tsunami deposition, providing an alternative means of event recognition [8–10].

Here we apply this molecular approach to the 2018 Palu–Donggala event to test whether microbial signatures can distinguish tsunami deposits from underlying sediments, extending previous applications from earthquake-triggered tsunami to landslide-triggered tsunamis.

We characterized microbial communities in sediment cores containing tsunami deposits overlying pre-tsunami layers, collected along the eastern (Pantoloan), western (Lolioge), and southern (Palu City) coast of Palu Bay, Central Sulawesi, Indonesia (Fig. 1). The reported tsunami inundation distance at Pantoloan is up to 275 m inland, with a run-up limit of ~5.5 m above mean sea level [11]. Similarly, the Palu City School site was inundated up to 270 m inland, with a run-up height between 2.8 and 7.8 m above mean sea level [11]. At Lolioge, the run-up height and inundation distance were not directly measured, though they are likely to range from ~3 to 6.4 m and roughly 110 m inland based on measurements to the north of the site by Omira *et al.* [12] and Widiyanto *et al.* [13]. Microbial communities were analyzed using 16S rRNA gene V6–V8 amplicon sequencing (online supplementary material). Nonmetric multidimensional scaling (NMDS) based on Bray–Curtis dissimilarity revealed distinct clustering patterns between tsunami and pre-tsunami samples across all the sites (Permutational Multivariate Analysis of Variance (PERMANOVA), *p* < .001; Supplementary Table 1), with no evidence that this pattern was driven by unequal dispersion (Permutational Analysis of Multivariate Dispersions (PERMDISP), *p* > .05; Supplementary Table 2), indicating that microbial assemblages effectively discriminate tsunami deposits from underlying pre-tsunami layers (Fig. 2).

**Figure 1:**
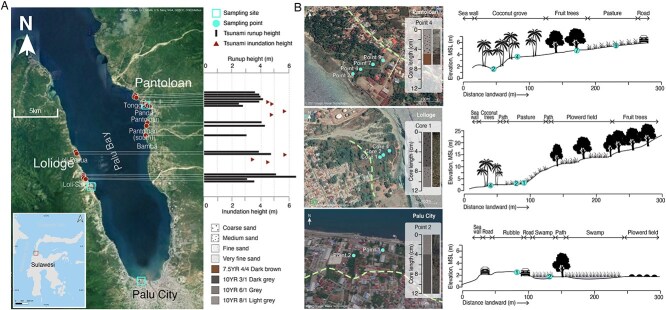
(A) Map of Palu Bay showing study sites (marked with blue boxes). The tsunami run-up heights and inundation heights of the 2018 Palu–Donggala tsunami are based on data from Omira *et al.* [12]. The inset shows the location of Palu Bay (marked with red box) within Central Sulawesi, Indonesia. (B) Close-up satellite images of three transects, Pantoloan (top), Lolioge (middle), and Palu City School (bottom). Sampling points are marked with blue circles (denoted as “Point” for Pantoloan and Palu City School transects and “Core” for the Lolioge transect). The inundation limit is marked by green dashed lines on the aerial photographs. The tsunami inundation limits for Pantoloan and Palu City were derived from Majewski *et al.* [11], whereas the limit for Lolioge was estimated based on field observations. Two of the transects, the Pantoloan and Palu City School transects, were previously reported in Majewski *et al.* [11], but samples for sedDNA analysis were only available at selected points, depending on the accommodation space; therefore, the sampling points doesn’t go in sequence. Representative sediment core lengths and stratigraphy are shown on the side of each satellite image. Simplified transect sketches based on field observations are provided to illustrate local topography and vegetation. Satellite imagery sourced from Google Earth with data providers as indicated on the figure.

**Figure 2:**
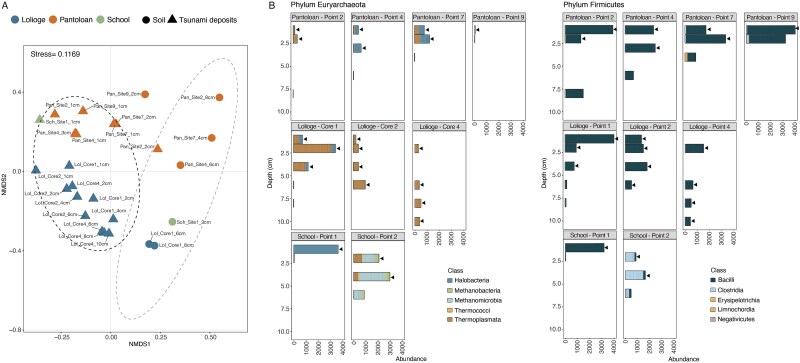
(A) NMDS based on Bray–Curtis dissimilarity matrix, visualizing microbial community differences between transects and sediment core layers sampled 2 months after the 2018 Palu tsunami. The ellipses represent 85% confident intervals, with the black ellipse grouping the tsunami deposits and the grey ellipse grouping the pre-tsunami layers. (B) Bar plots illustrating unique tsunami microbial signatures associated with the 2018 Palu tsunami, showing the relative abundance of taxa identified as differentially abundant between tsunami deposits and pre-tsunami layers (Supplementary Fig. 3). These tsunami-associated taxa are consistently present in tsunami deposits and absent or present at low abundances in pre-tsunami layers within the sediment cores. Tsunami deposits are marked with a black triangle. The left panel represents the phylum Euryarchaeota (Archaea), while the right panel represents the phylum Firmicutes (Bacteria). The bar plots are color-coded according to the associated phylogenetic class*.*

At Pantoloan, where sedimentological contrasts between tsunami and pre-tsunami layers were clear (Fig. 1; Supplementary Fig. 1), microbial profiles provided consistent evidence for tsunami identification (Fig. 2). At Lolioge, where grain-size differences were minimal (Fig. 1; Supplementary Fig. 1), microbial communities still showed clear differentiation between tsunami deposits and pre-tsunami layers (Fig. 2), highlighting the sensitivity of molecular indicators where conventional sedimentary evidence is limited. At the urban Palu City School site, tsunami sediments were characterized by taxa that were differentially abundant and commonly associated with organic-rich or anthropogenically influenced environments, including Methanomicrobia and Clostridia (Fig. 2) [14, 15]. Their increased abundance may reflect the transport and redistribution of microbially enriched sediments by the tsunami wave from the Palu River and bay. Nonetheless, the microbial assemblages at the Palu City School site tsunami deposit remained distinct from pre-tsunami layers (Figs 1 and 2). These findings underscore the robustness of sedDNA approaches in heterogeneous environments.

Differential abundance analysis identified 70 Amplicon Sequence Variants (ASVs) that were strongly associated with tsunami deposits; specifically Bacilli, Halobacteria, and Thermoplasmata were consistently associated with tsunami deposits across all the sites (Fig. 2; Supplementary Fig. 2). These taxa are tolerant of high salinity and environmental stress [16–18], suggesting that marine inundation and rapid geochemical shifts imposed strong selective pressures following the tsunami. Similar halotolerant taxa have been reported in overwash deposits from Japan [19] and Thailand [9], reinforcing the interpretation of marine inundation.

To infer community assembly processes, we used a null-model approach to examine the mechanisms driving microbial community assembly post-tsunami disturbance (online supplementary material) [20]. The analysis showed that the deterministic strength > 97% at Pantoloan and Lolioge, whereas the stochastic intensity remained <3% (Supplementary Table 3), indicating that community assembly was driven by predictable causal mechanisms (deterministic) rather than random or chaotic processes (stochastic).

Standardized effect sizes of beta diversity were strongly positive, confirming that observed community dissimilarities exceeded random expectations. These patterns suggest that pulse disturbances such as tsunamis drive community changes through environmental filtering, likely associated with marine water intrusion and the introduction of external resources that alter both abiotic conditions and biotic interactions [20]. Similar patterns were reported by Asano *et al.* [19], who reported that microbial community changes in tsunami deposits 1 year after inundation correlated with geochemical changes, highlighting the role of environmental filtering in structuring tsunami microbial assemblages.

Microbial community assembly in the tsunami deposits was dominated by deterministic processes across all the transects. The community composition nevertheless varied significantly among sites (pseudo-*F*_2,28_ = 3.4839, *P* = .0001; Supplementary Table 1), suggesting site-specific environmental filters. This heterogeneity explains the absence of a universal tsunami microbial signature across regions [8, 9, 21, 22]. Microbial responses post-tsunami disturbance reflect local environmental conditions, sedimentary settings, and the extent of inundation.

Over longer timescales, as new sediment layers accumulate and environmental conditions stabilize, deterministic control may weaken, while stochastic processes such as dispersal become more influential, especially in coastal regions with limited accommodation space [23]. This transition is supported by prior work showing that microbial differences between tsunami and background sediments diminish after several years [9], suggesting progressive ecological recovery [24, 25]. Nevertheless, when preserved in the sedimentary record, distinct microbial assemblages within tsunami layers can persist over centennial timescales [8, 9], providing valuable molecular evidence for past extreme flooding events.

Together, our findings demonstrate that sedDNA provides a sensitive and reliable tool for distinguishing tsunami deposits, even when physical sedimentary evidence is inconclusive. Microbial community shifts reflect deterministic assembly driven by environmental filtering immediately after inundation, emphasizing the site-specific nature of tsunami microbial signatures. While the specific taxa differentiating tsunami and pre-tsunami layers may vary by site, the consistent pattern of environmentally constrained microbial community restructuring supports the broader application of the molecular approach for identifying tsunami deposits in complex coastal settings. These results advance the molecular detection of extreme flooding events and highlight the value of integrating microbial and sedimentological proxies. As sequencing technologies advance with lower cost, and reference databases and bioinformatic tools expand and mature, such approaches will become increasingly powerful and accessible for reconstructing past events and assessing the ecological impacts of natural hazards across coastal environments.

## Supplementary Material

ISME_C_Brief_Communication_SI_revision_clean_final_ycag114

## Data Availability

The raw sequencing data that support the findings of this study have been deposited under Bioproject ID PRJNA752493. The processed data and scripts are available on https://github.com/wenshu-yap/2018PaluTsunami-microbes.git.
