# Supplementary material for: Microbial community shifts in deposits from the 2018 Palu–Donggala tsunami
Source: ISME Commun. 2026 Jul 3;6(1):ycag114. doi: 10.1093/ismeco/ycag114 (PMC13335651; doi:10.1093/ismeco/ycag114)
Supplement: ISME_C_Brief_Communication_SI_revision_clean_final_ycag114 [file isme_c_brief_communication_si_revision_clean_final_ycag114.docx]

**SUPPLEMENTARY INFORMATION**

**Microbial Community Shifts In Deposits From The 2018 Palu-Donggala Tsunami**

Wenshu Yap^a^*, Adam D. Switzer^a,b^, Jędrzej M. Majewski^a,c^, Winona Wijaya^b,d^, Ezequiel Santillan^e^, Rachel Y.S. Guan^b^, Benjamin P. Horton^f^, Benazir Benazir^g^, Ella Meilianda^h^, Federico M. Lauro^b,i^ .

^a^ Earth Observatory of Singapore, Nanyang Technological University, Singapore

^b^ Asian School of the Environment, Nanyang Technological University, Singapore

^c^ Institute of Geology, Adam Mickiewicz University, Poznań, Poland

^d^ Singapore Centre for Environmental Life Sciences Engineering, National University of Singapore, Singapore

^e^Singapore Centre for Environmental Life Sciences Engineering, Nanyang Technological University, Singapore

^f^ School of Energy and Environment, City University of Hong Kong, Hong Kong.

^g^ Civil and Environmental Engineering Department, Faculty of Engineering, Universitas Gadjah Mada, Jl. Grafika, Kampus No.2, Yogyakarta, Indonesia.

^h^ Tsunami and Disaster Mitigation Research Center, University Syiah Kuala, Indonesia

^i^ Luminis Water Technologies, Singapore

*Corresponding author

Email address: wenshu.yap@ntu.edu.sg

**Study Site Descriptions**

On 28 September 2018, a Mw 7.5 earthquake struck the Palu-Donggala region of Central Sulawesi, Indonesia, along the Palu-Koro strike-slip fault, generating a damaging tsunami within Palu Bay [1,2]. Post-event field surveys reported maximum tsunami run-up heights of 10.7 m at Tondo on the eastern coast [3] and 9.1 m at Benteng village on the western coast (Fig. 1) [4]. The tsunami Inundation depths showed a similar variation, ranging from 1.2 to 8.7 m, with the greatest depth again recorded at Benteng village (Fig. 1) [4]. In general, the inundation depth remained below 4 m along the western coast and between 3 to 5 m along the eastern coast further north (Fig. 1) [4]. This spatial gradient indicates that the southern part of Palu Bay, near its narrow end, experienced the most severe tsunami impact, with the intensity reduced toward the bay opening [3,4].

Palu Bay has a narrow, funnel-like morphology, with a broad entrance connecting to the Makassar Strait, and the morphology has been suggested to play a role in amplifying tsunami waves [5, 6]. The coastal landscape consists of steep sandy beaches and berms, that transition into relatively flat coastal plains, before rising sharply into mountainous terrain [4, 7]. Palu Bay is bounded by the Gawalise Mountains to the west and the Rovigadi Mountains to the east, both exceeding 2,000 m above sea level [7,8]. The bay has a maximum water depth of 830 m, with tidal variation ranging from 0.8 to 1.7 m [9].

Within this setting, our study focused on three locations along Palu Bay, selected to represent distinct sedimentary environments and coastal features (Fig. 1), as geomorphic setting influences tsunami impacts and sediment deposition [7]. We prioritized accessible areas with minimal disturbance from post-tsunami recovery operations. Pantoloan village, situated on the eastern coast, features a steep sandy beach which transitions into a gentle grassy slope, with coconut groves, fruit orchards, and pasture fields dominating the landscape [7]. In contrast, Lolioge village on the western coast is characterized by a steep, gravely to sandy coastline, primarily lined with coconut trees. The third site, Palu City, lies at the head of Palu Bay, and is marked by flat topography of former coastal wetlands, partly modified by urbanisation [7]. The sampling site at Palu City is located approximately 700 m west of the Palu River mouth, the main source of sediment discharge into Palu Bay [10].

**MATERIALS AND METHODS**

**Sedimentary environmental DNA (sedDNA) field sampling**

In November 2018, we sampled along transects on Palu Bay’s coastline, to map the inland distribution of sediments from the tsunami event. The sampling covered three locations: the eastern (Pantoloan), western (Lolioge) and southern coasts of Palu Bay (Palu City). Each transect extended from the shoreline to the landward inundation limit, where tsunami deposits were thinnest, covering a maximum distance of about 300 m. Elevations and distances of sampling points were measured with a Leica dumpy level, with detailed survey information reported in Majewski et al. [7]. Coordinates were recorded with a handheld GPS. We used a hand gouge auger to retrieve sediment cores to an average depth of 13 cm, while trowels were used to collect sediment samples ~~at~~ from shallow pits ~5 cm deep. Each core was subsampled at multiple depth intervals. The stratigraphy at each sampling point was recorded in the field, and detailed sedimentological characteristics were measured using laser diffraction particle size analysis. The category size (e.g., clay, very coarse silt, medium sand) and the logarithmic mean and sorting were calculated following Folk and Ward [11]. Due to accommodation space at the sites, we collected a total of 29 sediment samples from nine sampling points across the three locations for sedDNA analysis. This limitation explains why the sampling points shown in Figure 1 are not continuous.

To maintain sample integrity and minimize biological degradation during collection and processing, we followed the handling precautions outlined by Armbrecht et al. [12]. All sediment samples were collected in sterile 15 ml conical Falcon tubes and immediately stored in dry shippers filled with liquid nitrogen. All personnel wore powder-free surgical gloves during sample handling to reduce the risk of cross-contamination. Additionally, equipment, including the gouge auger and hand shovels, was pre-treated with a 20% bleach solution before sampling. All samples were transported to Singapore in dry shippers, and subsequently stored at −80 °C in an Ultra-Low Freezer facility upon arrival at the Asian School of the Environment, Nanyang Technological University, Singapore.

**DNA extraction and amplicon library construction**

We extracted DNA from 29 sediment samples using the PowerSoil DNA Isolation Kit (Qiagen, Hilden, Germany) with minor modifications. Initially, we added 200 μL of Phenol: Chloroform: Isoamyl-alcohol (25:24:1) and 60 μL Solution C1 into the 250 mg sediment samples within the bead beading tube. The samples are subjected to maximum speed vortex for 20min and followed with 28,000 g centrifugation for 1 min. Next, the supernatant is transferred into a new tube that contain 100 μL Solution C2 and 100 μL Solution C3. The mixture underwent brief vortexing and was incubated at 4 °C for 5 minutes. Subsequently, we centrifuged the mixture with the same setting and combined one part of Solution C4 with one part of the centrifuged supernatant, briefly vortexed this mixture, and incubated it at 4 °C for 5 minutes. We followed the manufacturer’s protocol for the subsequent steps until we obtained clean DNA as the final product. DNA quantities were measured using the Qubit dsDNA HS assay (Thermo Fisher Scientific, USA).

For the amplification of 16S rRNA genes through polymerase chain reactions (PCR), we used a universal primer set (926wF/1392R) that targets the 16S rRNA gene V6-V8 regions in archaea, bacteria, and eukaryotes [13,14]. Each PCR reaction contained 12.5 μl of 2x KAPA HiFi Hotstart Ready Mix (KAPA Biosystems, Cape Town, South Africa), 5 μl each of the forward and reverse primers (1 μM final concentration), and 2.5 μl of genomic DNA (5 ng/μL concentration). The amplification process entailed denaturation of the DNA at 95 °C for 3 minutes, followed by 20 cycles of amplification at 95 °C for 30 seconds, 55 °C for 30 seconds, and 72 °C for 30 seconds. The amplification concluded with a final extension step at 72 °C for 5 minutes to ensure completion of partially amplified products. To minimize potential PCR-based bias, we minimized the number of PCR cycles. Each sample was amplified in triplicate using a ThermoFisher SimpliAmp Thermal Cycler, combined, and purified using the Agencourt AMpure XP PCR purification system (Beckman Coulter, Singapore). The purified amplicons were quantified using the Qubit dsDNA HS assay. Finally, the amplicons were pooled and sequenced on an Illumina MiSeq system (version 3) with a 300 base-pairs paired-end read length by Macrogen Asia Pacific Pte Ltd (South Korea).

**16S rRNA gene bioinformatic processing**

The raw sequences underwent initially pre-processing, where we removed primers’ sequences using cutadapt (version 2.10) [15]. To ensure the high quality and accuracy of the sequences, we applied several filtering parameters. Specifically, we removed sequences that were shorter than 280 base pairs in the forward reads and 230 base pairs in reverse reads. Additionally, sequences with a maximum expected error rate, calculated using base calling error probabilities, exceeding two base pairs in the forward reads and five base pairs in reverse reads were also removed. These steps were carried out using the filterAndTrim function in the DADA2 package in R [16]. Following the removal of low-quality sequences, the sequences were corrected for errors the DADA2 algorithm [16]. Amplicon Sequence Variants (ASVs), which are formed based on DNA sequences differences, were then utilized to infer the true biological composition of the samples [16].

For taxonomic classification, we employed the Ribosomal Database Project (RDP) naïve Bayesian classifier [17] as implemented in the R DADA2 package, referencing the SILVA database (version 132) [18,19]. In the final database, we removed ASVs with a phylum-level bootstrap value below 90% to ensure high-confidence taxonomic classification. Additionally, ASVs with fewer than 10 counts were eliminated to minimize noise from low-abundance ASVs. The results of major microbial taxa (>1000) in each sampling sites are reported in Supplementary Figure 3.

**Statistical analysis**

Statistical comparison within and between samples was performed in the R version 3.6.1 statistical environment [20] using the phyloseq package [21]. The abundance data were normalized to the median sequencing depth before performing all the statistical analyses, except for alpha diversity. For alpha diversity, we transformed the abundance data into Hill number, which effectively express the number of species [22]. This approach overcome the non-linear scaling of traditional indices and allows direct comparison across communities. We report Hill numbers of order 1 and 2, which correspond to the Shannon diversity (H') and Simpson diversity, respectively.

We used Welch’s t-test to compare the alpha diversity variation between samples and the results are reported in Supplementary Table 4. Community differences between samples (beta-diversity) were visualized with non-metric multidimensional scaling (NMDS) ordination, based on Bray-Curtis dissimilarity matrix constructed from square-root transformed abundance data. We then tested for differences in community structure among groups using permutational multivariate analysis of variance (PERMANOVA) [23] and evaluated the homogeneity of within-group dispersion using permutational analysis of dispersion (PERMDISP) [24]. The complex environment and heavy anthropogenic influence in the Palu City School transect was notable when compared to the more natural setting at other sites. Therefore, sampling Point 2 from Palu City School transect was excluded from this analysis to minimize confusion.

As the sampling design was unbalanced depending on the accommodation space at each site, our dataset contained proportionally more tsunami samples than pre-tsunami samples. We took steps to ensure that our statistical tests were robust by applying two approaches. First, we down sampled the larger group (tsunami samples) to match the number of pre-tsunami samples, repeating this procedure 1,000 times. Second, we used bootstrapping with replacement to generate balanced replicates. We then repeated PERMANOVA and PERMDISP on these balanced datasets to confirm that results were not driven by uneven sampling. The result is presented in Supplementary Table 5.

A negative binomial generalized linear model was performed using DESeq 2 package [25] to examine the community changes before and after the tsunami disturbance. The significant model test was performed using the Wald test, and the p-value was corrected for multiple hypothesis testing [26].

We performed null-model analysis to test the ecological processes structuring microbial communities. This method compares observed how beta diversity deviates from values expected under random assembly [27]. In this analysis, ASVs were treated as species, and each read was considered an individual. To prevent the result from biasing due to uneven sample size, we used the down-sampled and bootstrapped dataset to run this analysis. We randomized the sample with ASVs count matrix while keeping row sums (sample totals) and column sums (ASVs totals) fixed. This is the classic fixed-fixed null using r2dtable based on Patefield algorithm [28]. Null models were constructed by randomizing the distribution of individuals among samples while preserving the number of individuals per sample, the relative abundance of each ASV, and the observed alpha diversity. For each dataset, we generated 10,000 randomizations to estimate the expected distribution of beta diversity. Standardized effect sizes (SES) and p-values were then derived by comparing observed beta diversity to this null distribution. Analyses were performed separately for the 2018 Palu tsunami deposits and the pre-tsunami layers.

**Supplementary Table 1** Multivariate analysis (PERMANOVA) assessing microbial community differences between two fixed factors: SamplingSite (Pantoloan transect vs. Lolioge transect vs. Palu City School transect) and SampleType (tsunami deposits vs. pre-tsunami samples). PERMANOVA was conducted using Bray-Curtis dissimilarities calculated on square-root transformed, normalized median ASV counts. A significant p-value (<0.05) is highlighted in bold.

|  | df | Sum of sqs | F model | R^2^ | p-value |
| --- | --- | --- | --- | --- | --- |
| SamplingSite | 2 | 2.2686 | 3.4839 | 0.2022 | **0.0001** |
| SampleType | 1 | 0.8099 | 2.4877 | 0.0722 | **0.0003** |
| Residuals | 25 | 8.1397 |  | 0.7256 |  |
| Total | 28 | 11.2182 |  | 1 |  |

**Supplementary *Table 2*** *Multivariate analysis to examine the homogeneity of dispersion (PERMDISP) between sampling site i.e. Pantoloan, Lolioge, and School transects and between sample type, i.e., tsunami deposits and pre-tsunami sediments*

A. Dispersion between Pantoloan transect, Lolioge transect, and School transect.

| Overall | df | Sum of Sqs | Mean Sq | F value | p-value |
| --- | --- | --- | --- | --- | --- |
| Groups | 2 | 0.0295 | 0.0147 | 2.2927 | 0.1211 |
| Residuals | 26 | 0.1673 | 0.0064 |  |  |

B. Dispersion between tsunami deposits and pre-tsunami sediments in the overall dataset.

| Overall | df | Sum of Sqs | Mean Sq | F value | p-value |
| --- | --- | --- | --- | --- | --- |
| Groups | 1 | 0.0030 | 0.0030 | 0.5475 | 0.4672 |
| Residuals | 27 | 0.1503 | 0.0056 |  |  |

C. Dispersion between tsunami deposits and pre-tsunami sediment in Pantoloan transect.

| Pantoloan | df | Sum of Sqs | Mean Sq | F value | p-value |
| --- | --- | --- | --- | --- | --- |
| Groups | 1 | 0.0022 | 0.0022 | 0.3034 | 0.5952 |
| Residuals | 9 | 0.0669 | 0.0074 |  |  |

D. Dispersion between tsunami deposits and pre-tsunami sediment in Lolioge transect.

| Lolioge | df | Sum of Sqs | Mean Sq | F value | p-value |
| --- | --- | --- | --- | --- | --- |
| Groups | 1 | 0.1117 | 0.1117 | 52.4450 | 0.00002 |
| Residuals | 11 | 0.0234 | 0.00213 |  |  |

E. Dispersion between tsunami deposits and pre-tsunami sediment in School transect.

| School | df | Sum of Sqs | Mean Sq | F value | p-value |
| --- | --- | --- | --- | --- | --- |
| Groups | 1 | 0.0002 | 0.0002 | 0.0094 | 0.9289 |
| Residuals | 3 | 0.0763 | 0.0254 |  |  |

***
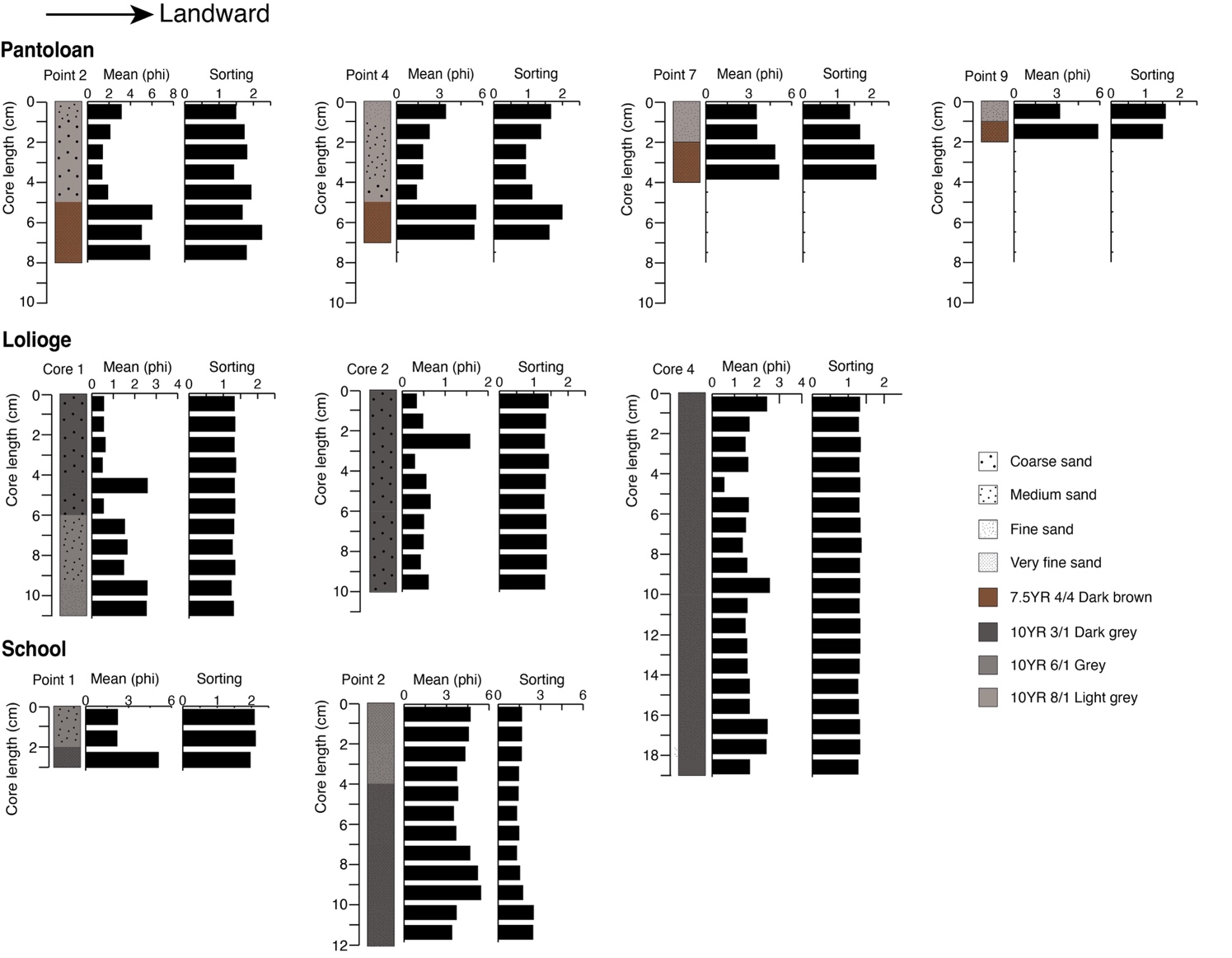
***

***Supplementary Figure 1*** *Grain size mean (phi) and sorting of Pantoloan, Lolioge and Palu City School transect.*


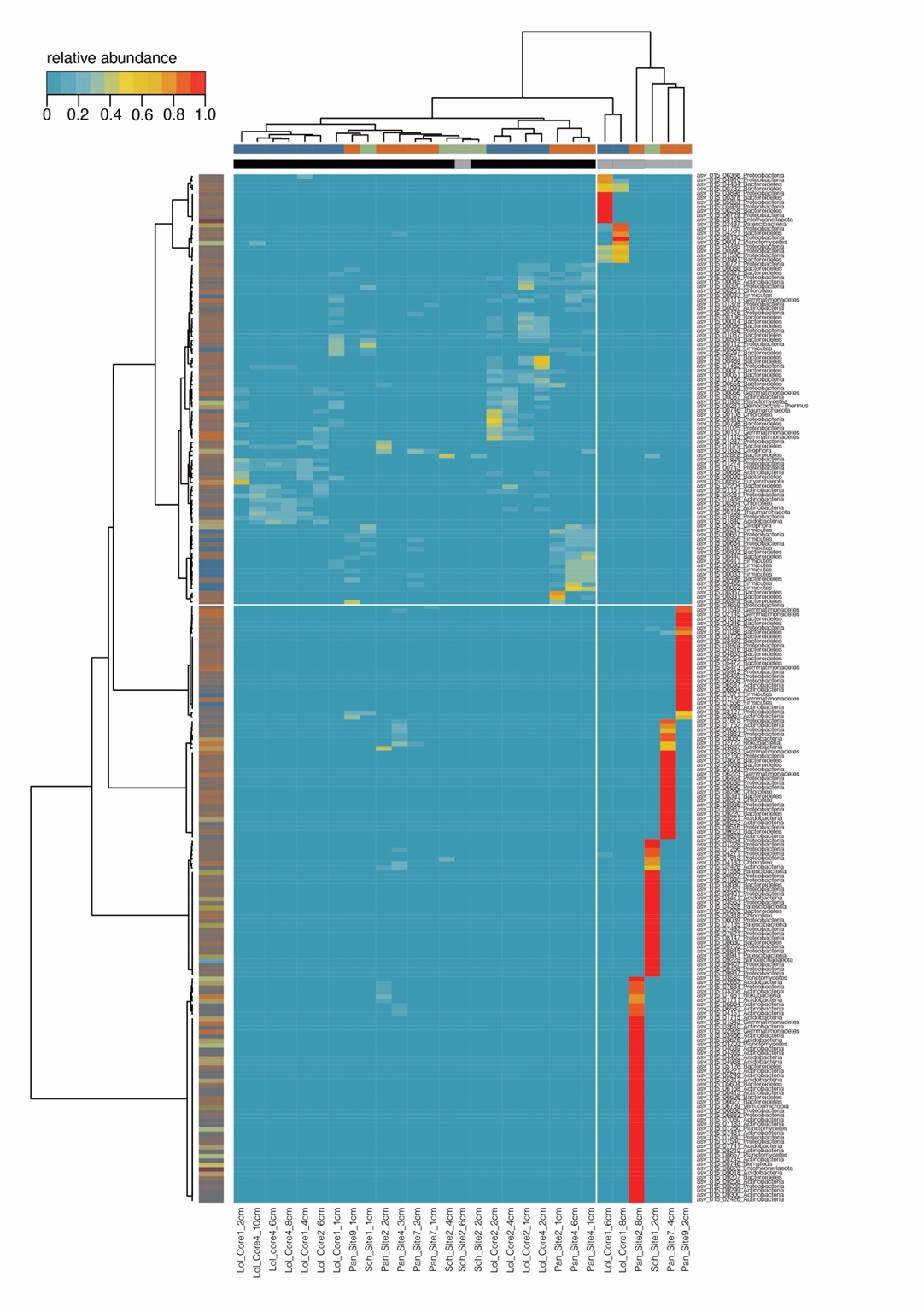


***Supplementary Figure 2*** *Heatmap presenting 70 taxa (after the white vertical line) that were differentially abundant in the tsunami sediments (indicated as grey bar in the second row after the vertical hierarchical analysis) but missing or low abundant in tsunami deposits (indicated as black bar in the second rows after the horizontal hierarchical analysis) determined using a negative binomial generalized linear model. The phylogenetic identification of each ASVs were provided at phylum level. The vertical hierarchical analysis is colour coded based on the SamplingSite (Lolioge transect – blue, Pantoloan transect – orange, School transect – green) and SampleType (tsunami deposits – black; pre-tsunami sediments – grey). Detailed samples’ description is listed at the end of the heatmap.*

***Supplementary Figure 3*** *Abundance of major microbial taxa (>1000) across sampling sites. Stacked bar plots illustrate the taxonomic composition of microbial communities at the phylum level based on 16S rRNA gene V6-V8 amplicon sequencing. Colors indicate different phyla.*

***Supplementary Table 3*** *Null model results for microbial community assembly in tsunami deposits and pre-tsunami layers from Lolioge and Pantoloan. (a) dataset resampled by downsampling and (b)dataset across 1,000 bootstrap replicates. Deterministic strength (DS) and stochastic intensity (SI) are expressed as percentages, standard effect size (SES) quantifies the deviation of observed beta-diversity from null expectation, and the beta ratio represent the ratio of mean null beta to observed beta. Values represent the median and 95 percentile range (2.5 – 97.5) are reported for bootstrap dataset. The number of samples per group after resampling is 4 per site per sample type.*

1. Downsampling dataset with no replacement

|  |  | DS (%) | SI (%) | SES | Beta ratio |
| --- | --- | --- | --- | --- | --- |
| Lolioge | 2018 Palu Tsunami deposits | 96.37 | 3.63 | 508.97 | 0.04 |
|  | Pre-tsunami layers | 96.99 | 3.01 | 533.75 | 0.03 |
| Pantoloan | 2018 Palu Tsunami deposits | 96.68 | 3.32 | 509.33 | 0.03 |
|  | Pre-tsunami layers | 97.33 | 2.67 | 626.19 | 0.03 |

1. Bootstrapping dataset with replacement

|  |  | DS (%)  Median  (2.5-97.5) | SI (%)  Median  (2.5-97.5) | SES  Median  (2.5-97.5) | Beta ratio  Median  (2.5-97.5) |
| --- | --- | --- | --- | --- | --- |
| Lolioge | 2018 Palu Tsunami deposits | 96.40 (95.74 – 97.52) | 3.60 (2.48 - 4.26) | 491.81 (369.40 – 563.40) | 0.04 (0.03 – 0.04) |
|  | Pre-tsunami layers | 97.37 (96.67 – 99.09) | 2.63 (0.90 – 3.33) | 489.29 (259.35 – 628.35) | 0.03 (0.01 – 0.03) |
| Pantoloan | 2018 Palu Tsunami deposits | 96.60 (95.84 – 98.38) | 3.40 (1.62 – 4.16) | 486.23 (319.07 – 586.74) | 0.03 (0.02 – 0.04) |
|  | Pre-tsunami layers | 97.48 (96.54 – 99.02) | 2.52 (0.98 – 3.46) | 569.35 (339.96 – 684.83) | 0.03 (0.01 – 0.04) |

**Supplementary *Table 4*** *Summary of the total number of sediment samples collected from each location and the corresponding Amplicon Sequence Variance (ASV) counts. The alpha diversity within each location is represented using Simpson and Shannon indices, with mean and standard deviation (sd) values indicated.*

|  | Pantoloan | Lolioge | School |
| --- | --- | --- | --- |
| Total samples numbers | 11 | 13 | 5 |
| Sum of ASV counts | 4,415 | 4,891 | 2,894 |
| Shannon (tsunami \| pre-tsunami) : | | |  |
| Mean | 313.12 \| 373.74 | 463.09 \| 373.41 | 380.41 \| 365.94 |
| Standard deviation | 69.06 \| 107.43 | 154.77 \| 137.03 | 192.02 \| 131.58 |
| Welch t-test p-value | 0.3620 | 0.5155 | n/a |
| Simpson (tsunami \| pre-tsunami) : | | | |
| Mean | 128.67 \| 172.45 | 222.08 \| 113.51 | 226.56 \| 191.67 |
| Standard deviation | 56.75 \| 78.95 | 105.75 \| 91.94 | 191.38 \| 174.10 |
| Welch t-test p-value | 0.3761 | 0.3073 | n/a |

***Supplementary Table 5*** *Results of Multivariate analysis on balanced datasets generated by (a) downsampling and (b) bootstrapping. PERMANOVA was used to test for differences in microbial community composition, and PERMDISP was used to test for homogeneity of dispersion. Analyses were performed across sampling site i.e. Pantoloan, Lolioge, and School transects and between sample types, i.e., tsunami deposits and pre-tsunami sediments*

1. Downsampling dataset with no replacement

| PERMANOVA | df | Sum of sqs | F model | R^2^ | p-value |
| --- | --- | --- | --- | --- | --- |
| SamplingSite | 2 | 1.7067 | 0.2396 | 2.6703 | **0.001** |
| SampleType | 1 | 0.5513 | 0.0774 | 1.7250 | **0.020** |
| SamplingSite:SampleType | 2 | 1.0317 | 0.1448 | 1.6143 | **0.010** |
| Residuals | 12 | 3.8348 |  | 0.5383 |  |
| Total | 17 | 7.1244 |  | 1 |  |

| PERMDISP | df | Sum of Sqs | Mean Sq | F value | p-value |
| --- | --- | --- | --- | --- | --- |
| Groups | 1 | 0.0089 | 0.0089 | 1.3066 | 0.269 |
| Residuals | 16 | 0.1093 | 0.0068 |  |  |

1. Bootstrapping dataset with replacement (500 replicates)

| PERMANOVA | df | Sum of sqs | F model | R^2^ | p-value |
| --- | --- | --- | --- | --- | --- |
| SamplingSite | 1 | 1.2269 | 0.2144 | 4.7415 | **0.001** |
| SampleType | 1 | 0.6102 | 0.1066 | 2.3583 | **0.012** |
| SamplingSite:SampleType | 1 | 0.7801 | 0.1363 | 3.0147 | **0.001** |
| Residuals | 12 | 3.1052 |  | 0.5426 |  |
| Total | 15 | 5.7225 |  | 1 |  |

| PERMDISP | df | Sum of Sqs | Mean Sq | F value | p-value |
| --- | --- | --- | --- | --- | --- |
| Groups | 1 | 0.00026 | 0.00026 | 0.0106 | 0.926 |
| Residuals | 14 | 0.3430 | 0.0245 |  |  |

**REFERENCES**

| 1 |  | Fang J, Xu C, Wen Y, et al. The 2018 Ms 7.5 Palu Earthquake: a supershear rupture event constrained by InSAR and broadband regional seismograms. *Remote Sens* 2019;**11**(11):1330. https://doi.org/10.3390/rs11111330 |
| --- | --- | --- |
| 2 |  | Heidarzadeh M, Muhari A, Wijanarto AB. Insight on the source of the 28 September 2018 Sulawesi Tsunami, Indonesia based on spectral analyses and numerical simulations. *Pure Appl Geophys* 2019;**176**:25-43. https://doi.org/10.1007/s00024-018-2065-9 |
| 3 |  | Widiyanto W, Santoso PB, Hsiao SC, et al. Post-event field survey of 28 September 2018 Sulawesi earthquake and tsunami. *Nat Hazards Earth Syst Sci* 2019;**1**:1-23. https://doi.org/10.5194/nhess-19-2781-2019 |
| 4 |  | Omira R, Dogan GG, Hidayat R, et al. The September 28th, 2018, Tsunami in Palu-Sulawesi, Indonesia: A post-event field survey. *Pure Appl Geophys* 2019;**176**:1379-1395. https://doi.org/10.1007/s00024-019-02145-z |
| 5 |  | Carvajal M, Araya-Cornejo C, Sepúlveda I, et al. Nearly instantaneous tsunamis following the Mw 7.5 2018 Palu earthquake. *Geophys Res Letts* 2019;**46**(10):5117-5126. <https://doi.org/10.1029/2019GL082578> |
| 6 |  | Antariksa M, Ishak MG, Tunas IG. Analysis of changes in bathymetry of the Palu River estuary and its effect on flow characteristics. *Int J Adv Sci Technol* 2020;**29**(4):6195-6208. |
| 7 |  | Majewski JM, Switzer AD, Guan RYS, et al. Sediment analysis and historical context of the 2018 Palu-Donggala tsunami deposit, Indonesia. *Mar Geol* 2023;**466**(107159). https://doi.org/10.1016/j.margeo.2023.107159 |
| 8 |  | Patria A, Putra PS. Development of the Palu-Koro fault in NW Palu valley, Indonesia. *Geosci Lett* 2020:**7**(1):1-11. https://doi.org/10.1186/s40562-020-0150-2 |
| 9 |  | TidesChart. Tide Times and Tide Chart for Palu. https://www.tideschart.com/Indonesia/Central-Sulawesi/Palu/ (17 May 2025, date last accessed). |
| 10 |  | Frederik MC, Udrekh, Adhitama R, et al. First results of a bathymetric survey of Palu Bay, Central Sulawesi, Indonesia following the Tsunamigenic Earthquake of 28 September 2018. *Pure Appl Geophys* 2019;**176**(8):3277-3290. https://doi.org/10.1007/s00024-019-02280-7 |
| 11 |  | Folk RL, Ward WC. Brazos River bar: a study in the significance of grain size parameters. *J Sediment Petrol* 1957;**27**(1):3-26. https://doi.org/10.1306/74D70646-2B21-11D7-8648000102C1865D |
| 12 |  | Armbrecht LH, Coolen MJL, Lejzerowicz F, et al. Ancient DNA from marine sediments: precautions and considerations for seafloor coring, sample handling and data generation. *Earth-Sci Rev* 2019;**196**:102887. https://doi.org/10.1016/j.earscirev.2019.102887 |
| 13 |  | Wilkins D, Sebille E, Rintoul SR, et al. Advection shapes Southern Ocean microbial assemblages independent of distance and environment effects. *Nat Commun* 2013;**4**(2457):1-7. https://doi.org/10.1038/ncomms3457 |
| 14 |  | Allen MA, Cavicchioli R. Microbial communities of aquatic environments on Heard Island characterized by pyrotag sequencing and environmental data. *Sci Rep* 2017;**7**:1–16. https://doi.org/10.1038/srep44480 |
| 15 |  | Martin M. Cutadapt removes adapter sequences from high-throughput sequencing reads. *EMBnet* 2011;**17**(1):10-12. https://doi.org/10.14806/ej.17.1.200 |
| 16 |  | Callahan BJ. DADA2: high-resolution sample inference from Illumina amplicon data. *Nat Methods* 2016;**13**(7):581-583. https://doi.org/10.1038/nmeth.3869 |
| 17 |  | Wang Q, Garrity GM, Tiedje JM, et al. Naïve Bayesian classifier for rapid assignment of rRNA sequences into the new bacterial taxonomy. *Appl Environ Microbiol* 2007;**73**(16):5261-5267. https://doi.org/10.1128/AEM.00062-07 |
| 18 |  | Quast C, Pruesse E, Yilmaz. P, et al. The SILVA ribosomal RNA gene database project: improved data processing and web-based tools. *Nucleic Acids Res* 2012;**41**(D1):D590-D596. https://doi.org/10.1093/nar/gks1219. |
| 19 |  | Yilmaz P, Parfrey LW, Yarza P, et al. The SILVA and "All-species Living Tree Project (LTP)" taxonomic frameworks. *Nucleic Acids Res* 2014;**42**(Database issue):D643-8. https://doi.org/10.1093/nar/gkt1209 |
| 20 |  | R Core Team. R: A language and environment for statistical computing. Vienna: R Foundation for Statistical Computing 2021. https://www.R-project.org/ |
| 21 |  | McMurdie PJ, Holmes S. phyloseq: An R Package for Reproducible Interactive Analysis and Graphics of Microbiome Census Data. *PLoS ONE* 2013;**8**(4):e61217. https://doi.org/10.1371/journal.pone.0061217 |
| 22 |  | Jost L. Entropy and diversity. *Oikos* 2006;**113**(2):363-375. https://doi.org/10.1111/j.2006.0030-1299.14714.x |
| 23 |  | Anderson MJ. A new method for non-parametric multivariate analysis of variance. *Austral Ecol* 2008;**26**:32–46. https://doi.org/10.1111/j.1442-9993.2001.01070.pp.x |
| 24 |  | Anderson MJ. Distance-based tests for homogeneity of multivariate dispersion. *Biometrics* 2006;**62**(1):245-253. https://doi.org/10.1111/j.1541-0420.2005.00440.x |
| 25 |  | Love MI, Huber W, Anders S. Moderated estimation of fold change and dispersion for RNA-seq data with DESeq2. *Genome Biol* 2014;**15**(12):1-21. https://doi.org/10.1186/s13059-014-0550-8 |
| 26 |  | Benjamini Y, Hochberg Y. Controlling the false discovery rate: a practical and powerful approach to multiple testing. *J Royal Stat: Series B (Methodological)*, 1995;**57**(1):289-300. http://www.jstor.org/stable/2346101. |
| 27 |  | Santillan E, Constancias F, Wuertz S. Press disturbance alters community structure and assembly mechanisms of bacterial taxa and functional genes in mesocosm-scale bioreactors. *mSyst* 2020;**5**(4):e00471-20. https://doi.org/10.1128/mSystems00471-20. |
| 28 |  | Patefield WM. Algorithm AS 159: An efficient method of generating random R x C tables with given row and column totals. *Appl Stat* 1981;**30**(1):91. https://doi.org/10.2307/2346669 |
